# Chloroplast characterizations of a *Phalaenopsis* native to China, *Phalaenopsis mannii* (Orchidaceae)

**DOI:** 10.1080/23802359.2020.1833776

**Published:** 2020-11-11

**Authors:** Bin Chen, Yanping Zhang, Yinghui Cao, Yan Zheng, Ziling Wei, Kai Zhao, Donghui Peng, Yuzhen Zhou

**Affiliations:** a College of Landscape Architecture, Fujian Agriculture and Forestry University, Fuzhou, China; b College of Life Sciences, Fujian Normal University, Fuzhou, China

**Keywords:** *Phalaenopsis mannii*, chloroplast genome, phylogenetics

## Abstract

*Phalaenopsis mannii*, one of the native *Phalaenopsis* in China, is an important parent for breeding new varieties. However, its position has been unclear in *Phalaenopsis*. The obtained high-quality *P. mannii* chloroplast genome will provide useful information for phylogenetic and future breeding of *Phalaenopsis*. Herein, we reported a complete chloroplast genome of *P. mannii* from Yunnan, China. The sequencing data obtained from BGISEQ-500 platform were assembled. This sequence had a circular molecular length of 148,596 bp and contained a total of 127 genes with an average GC content of 36.7%. Phylogenetic analysis indicated that *Phalaenopsis* was monophyletic with strong support, in which the *P.mannii* was the sister-group of *Phalaenopsis aphrodite* subsp. formosas, *Phalaenopsis* ‘TinyStar’ and *Phalaenopsis equestris*.

*Phalaenopsis*, as a member of orchid family, is famous for its high ornamental characteristics and unique taxonomic status. It is also highly regarded by many scholars. However, the taxonomic position of some species in *Phalaenopsis* are still unclear. *Phalaenopsis mannii*, which is used as an important parent in cross breeding, is an epiphytic orchid native to southern Yunnan province, China (Chen and Ji 2000). Previously, largely according to flower and other morphological characteristics, *P. mannii* belongs to the sect. *Polychilos*. But phylogenetic trees constructed based on nuclear or chloroplast genes manifested that *P. mannii* was embedded in sect. *Amboinenses,* and sometimes it was the sister relationship to sect. *Amboinenses* and sect. *Zebrinae* as a single branch (Ito et al. 2005; Tsai et al. 2010). Thus, it is interesting to assemble and characterize the *P. mannii* chloroplast genome for providing a better studying phylogenetic and future breeding of *Phalaenopsis*.

Fresh samples of *P. mannii* was collected from Yunnan province, China (23°12′N, 104°70′E), and the living plants deposited in Fujian Agriculture and Forestry University (26°04′51.3″N, 119°14′19.9″E, Voucher specimen FAFU: HDL-YN2019-12A). Total genomic DNA from fresh *P. mannii* leaves was extracted with the DP305-Plant Tissues Genome DNA Extraction Kit (TianGen, Beijing, China) according to the kit instructions. The DNA sheared to approximately 300-500 bp. Then the libraries were constructed through end-repair, A-tailing, adapter ligation. Approximately 20 Gb clean reads were obtained through the BGISEQ-500 platform sequenced. After adapters and low-quality reads were removed by fastp software (Mak et al. 2017; Chen et al. 2018). Draft chloroplast genome was obtained using SPAdes v 3.13.1 software and manually corrected using Bandage v 0.8.1 software (Zhou et al. 2019). The assembled chloroplast genome was annotated by the online tool Geseq (Tillich et al. 2017) and Geneious v 2020.2.1 software (reference: *P.* ‘Tiny Star’, KJ944326) and then checked manually. In the end, we established the complete chloroplast genome sequence of *P. mannii* (GenBank accession MT822270) with a circular molecule of 148,596 bp in length and overall GC content of 36.7%. This chloroplast genome included an LSC region of 85,300 bp, an SSC region of 11,640 bp, and a pair of inverted repeats regions of 25,828 bp. We annotated 127 genes for the chloroplast genome, including 76 protein-coding genes (PCG), 37 transfer RNAs (tRNAs), eight ribosomal RNA (rRNA) genes, and six pseudogenes.

The phylogenetic tree was constructed to demonstrate the relationships between *Phalaenopsis* and other genera. The *P.mannii* chloroplast genome was aligned with other 24 *Aeridinae* chloroplast genome sequences and three *Orchidinae* species as the outgroup by Mafft v 7.455, and the construct the maximum likelihood tree by RAxML v 8.2.12 (Katoh and Standley 2013; Stamatakis 2014). All the sequences were downloaded from NCBI GenBank. The maximum likelihood tree indicated that *Phalaenopsis* formed a monophyletic clade, in which the *P.mannii* was the sister-group of (*Phalaenopsis equestris,* (*Phalaenopsis aphrodite* subsp. formosas*, Phalaenopsis* ‘TinyStar’)) (Figure 1).

**Figure 1.**
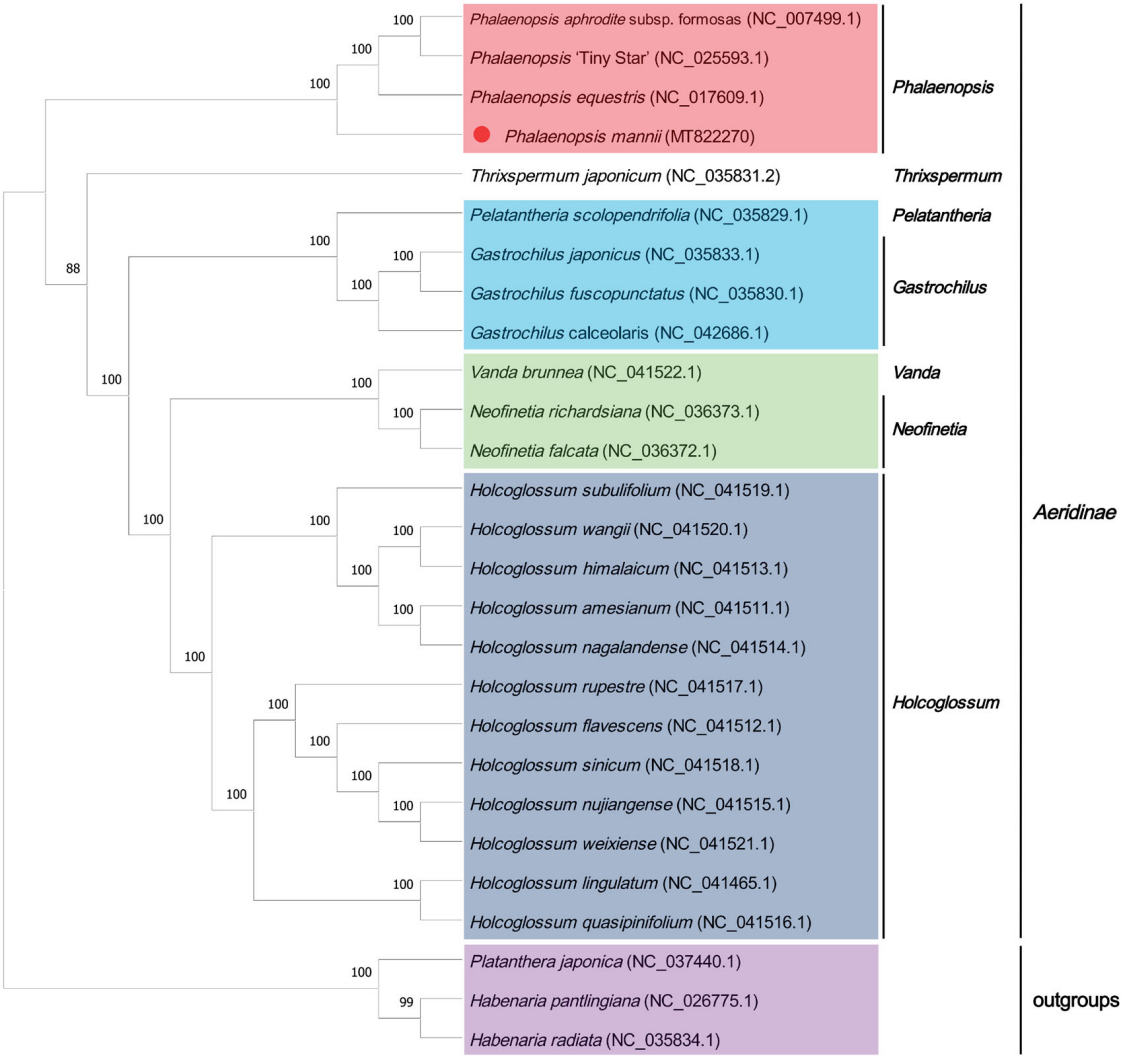
Maximum likelihood tree of 24 chloroplast sequences in *Aeridinae*, with *Platanthera japonica*, *Habenaria pantlingiana* and *Habenaria radiata* as outgroups. *Phalaenopsis mannii* was marked with a red circle. Bootstrap support values are shown next to the nodes.

## Data Availability

The raw data has been stored in omics database of Genome Sequence Archive. GSA accession number is CRA003144. All the information can be found on the website (https://bigd.big.ac.cn/gsa/browse/CRA003144/CRX150834).
